# Traumatic Brain Injury and Chronic Traumatic Encephalopathy: Not Only Trigger for Neurodegeneration but Also for Cerebral Amyloid Angiopathy?

**DOI:** 10.3390/biomedicines13040881

**Published:** 2025-04-05

**Authors:** Marialuisa Zedde, Fabrizio Piazza, Rosario Pascarella

**Affiliations:** 1 Neurology Unit, Stroke Unit, Azienda Unità Sanitaria Locale-IRCCS di Reggio Emilia, Viale Risorgimento 80, 42123 Reggio Emilia, Italy; 2 CAA and AD Translational Research and Biomarkers Lab, School of Medicine, University of Milano-Bicocca, 20900 Monza, Italy; fabrizio.piazza@unimib.it (F.P.); rosario.pascarella@aulss5.veneto.it (R.P.); 3 Neuroradiology Unit, Ospedale Santa Maria della Misericordia, AULSS 5 Polesana, 45100 Rovigo, Italy; 4 iCAβ International Network; 5 SINdem Study Group “The Inflammatory Cerebral Amyloid Angiopathy and Alzheimer’s Disease Biomarkers”

**Keywords:** traumatic brain injury, cerebral amyloid angiopathy, AD, chronic traumatic encephalopathy, neurodegeneration, inflammation, amyloid-beta, MRI

## Abstract

Traumatic brain injury (TBI) has been linked to the development of neurodegenerative diseases, particularly Alzheimer’s disease (AD) and chronic traumatic encephalopathy (CTE). This review critically assesses the relationship between TBI and cerebral amyloid angiopathy (CAA), highlighting the complexities of diagnosing CAA in the context of prior head trauma. While TBI has been shown to facilitate the accumulation of amyloid plaques and tau pathology, the interplay between neurodegenerative processes and vascular contributions remains underexplored. Epidemiological studies indicate that TBI increases the risk of various dementias, not solely AD, emphasizing the need for a comprehensive understanding of TBI-related neurodegeneration as a polypathological condition. This review further delineates the mechanisms by which TBI can lead to CAA, particularly focusing on the vascular changes that occur post-injury. It discusses the challenges associated with diagnosing CAA after TBI, particularly due to the overlapping symptoms and pathologies that complicate clinical evaluations. Notably, this review includes a clinical case that exemplifies the diagnostic challenges posed by TBI in patients with subsequent cognitive decline and vascular pathology. By synthesizing current research on TBI, CAA, and associated neurodegenerative conditions, this review aims to foster a more nuanced understanding of how these conditions interact and contribute to long-term cognitive outcomes. The findings underscore the importance of developing standardized diagnostic criteria and imaging techniques to better elucidate the relationship between TBI and vascular pathology, which could enhance clinical interventions and inform therapeutic strategies for affected individuals.

## 1. Introduction

Traumatic brain injury (TBI) research in humans has revealed the development of amyloid plaques following a severe single TBI and tau pathology after repeated mild TBIs (mTBI) [1,2,3]. These findings have led to the theory that a single moderate-to-severe TBI could elevate the risk of Alzheimer’s disease (AD) later in life, while repeated mild TBIs might increase the likelihood of chronic traumatic encephalopathy (CTE). In this review, we critically examine this hypothesis by looking at epidemiological and case–control studies, neuropathological evidence, and animal research. Epidemiological research highlights that TBI is linked to a greater risk of various types of dementia, not just AD, and can also lead to other neurodegenerative conditions, including Parkinson’s disease [4]. Moreover, postmortem studies on individuals who have experienced either a single TBI or repeated mild TBIs reveal a mix of amyloid, tau, TDP-43, and Lewy body pathologies [5,6,7]. This suggests that the brain damage caused by TBI is best understood as a “polypathology” rather than a single disease process. Preclinical studies back this up, showing that multiple proteins tied to neurodegenerative diseases accumulate in the brain following TBI [8,9].

The long-term effects of both a single TBI and repeated mild TBIs share common neuropathological features and clinical symptoms similar to those seen in traditional neurodegenerative diseases. However, while chronic cognitive and behavioral symptoms following repeated mTBI are often linked to CTE, similar symptoms following a single TBI are generally considered to be part of diseases like AD. These findings support the idea that all TBI-related neurodegenerative disorders, regardless of the nature or frequency of injury, should be grouped together under the broader category of traumatic encephalopathy or trauma-induced neurodegeneration [10].

While the topic of TBI accelerating or triggering a degenerative condition is well described, the relationship with the vascular component of amyloid-related pathology, namely cerebral amyloid angiopathy (CAA), has been much less studied. This occurs for several reasons—first of all, the fact that the in vivo diagnosis of CAA is based on a specific neuroradiological semeiology that requires the exclusion of altering causes of microhemorrhagic marathons and TBI is an alternative cause that in itself excludes the diagnosis of CAA [11]. Therefore, there is little evidence of the association between TBI and CAA, and research conducted on the iatrogenic variant of CAA has called this topic into question [12,13]. In fact, among the triggers of iatrogenic CAA, neurosurgical procedures commonly performed following severe TBI have been described, not only with dural graft implantation. Growing evidence links prolonged contact-sport exposure and RHI to various neurodegenerative diseases [14,15,16,17,18,19]. However, its role in small-vessel disease remains unclear.

The aim of this review is to examine the mechanisms and possible associations between TBI and CAA, particularly from the perspective of putative biological mechanisms and neuroradiological and pathological data.

## 2. Definitions: Traumatic Brain Injury and Traumatic Brain Injury-Induced Encephalopathy

The long-term consequences of repeated mild traumatic brain injuries (mTBI) in sports have been recognized since the 1920s [20], yet the link between a single TBI and dementia remains an area of ongoing investigation. Each year, approximately four million TBIs occur in the United States, and a majority of them are classified as mild [8]. The effects of head trauma—both immediate and chronic—have been documented for nearly a century. In 1927, “traumatic encephalitis” was used to describe lingering post-concussion symptoms in over 100 clinical cases [21]. Shortly after, the term “punch-drunk” was coined to characterize professional boxers experiencing symptoms such as ataxia, disorientation, and clumsiness [20]. While many cases were mild, severe instances displayed symptoms resembling Parkinson’s disease and dementia. By 1937, “dementia pugilistica” was introduced to describe the cognitive decline seen in boxers [22], evolving into “chronic post-traumatic encephalopathy” by the late 1950s [23]. Today, the condition is widely known as chronic traumatic encephalopathy (CTE), a term used to define the neuropathological damage caused by repeated concussive or subconcussive impacts, extending beyond the realm of boxing to include athletes, soldiers, and civilians. Among approximately 160 pathological cases of CTE across various populations with a history of repeated mTBIs [24], the neuropathology of CTE was described, but its clinical presentation remains incompletely understood. Individuals with confirmed CTE often exhibit a complex spectrum of symptoms overlapping with neurodegenerative diseases, such as AD, frontotemporal dementia (FTD), Parkinson’s disease (PD), and amyotrophic lateral sclerosis (ALS) [15].

CTE is an advancing neurodegenerative condition typically triggered by repeated head injuries. In some instances, CTE may develop following a singular moderate-to-severe TBI that, for reasons yet to be fully understood, gradually leads to dementia. This condition, initially recognized in 1928 among boxers referred to as “punch drunk”, was later termed “dementia pugilistica” (DP) [25]. Over time, it became clear that athletes from various sports were experiencing similar forms of dementia, prompting the renaming of DP to “chronic traumatic encephalopathy” (CTE) during the 1960s. As highlighted by Mckee and Daneshvar [26], the prominent pathological characteristics of CTE include widespread cerebral atrophy, affecting the frontal and temporal lobes, as well as atrophy in areas like the thalamus, hypothalamus, and mammillary bodies—regions linked to memory recall. Additional features include an enlargement of the lateral and third ventricles and a thinning of the corpus callosum, accompanied by a pallid appearance of the substantia nigra and locus coeruleus. In 2016, a consensus conference was convened to establish neuropathological criteria for diagnosing CTE. The conclusions of this meeting, documented by McKee et al. [27], defined CTE as a neurodegenerative disorder characterized by the abnormal build-up of hyperphosphorylated tau protein in the brain. It was recognized that “currently, a definitive diagnosis of CTE can only be made through post-mortem examination of brain tissue”. The hallmark lesion of CTE was identified as an accumulation of hyperphosphorylated tau (p-tau) in neurons and astroglia situated around small blood vessels in the depths of cortical sulci in an irregular distribution. Supportive, albeit non-specific, features of CTE include pre-tangles and neurofibrillary tangles (NFTs) affecting the superficial layers of the cerebral cortex, with additional pre-tangles, NFTs, or extracellular tangles in CA2, and pre-tangles and proximal dendritic swellings in CA4 of the hippocampus. Perl [28] notes that tau is the primary component of neurofibrillary tangles, with other proteins, such as ubiquitin, cholinesterases, and amyloid-beta 4 (Ab4), present, but tau remains the critical element. In a recent review on tau-related neurodegenerative diseases, Josephs [29] begins with insights into tau biology, emphasizing that tau is a microtubule-associated protein crucial for stabilizing and assembling microtubules, which are vital for effective axonal transport. In a healthy brain, tau is primarily located within neurons and predominantly within axons. Josephs explains that the tau amino acid sequence can be categorized into four domains: (i) the N-terminal domain, (ii) a proline-rich domain, (iii) a microtubule-binding domain, and (iv) the C-terminal domain. “In its usual state, tau is unfolded and phosphorylated, while its abnormal form, found in the brains of patients with primary tauopathies, is marked by hyperphosphorylated and aggregated tau. The attachment of tau to microtubules is governed by the equilibrium between phosphorylation and dephosphorylation of tau”. Issues related to hyperphosphorylated tau may stem from an increased proportion of phosphorylated tau sequences, surpassing the rise in the number of phosphorylated epitopes per sequence. Josephs [29] describes CTE as a “primary tauopathy consisting of mixed 3-repeat and 4-repeat tau isoforms”. He states, “There are six tau isoforms expressed in the adult brain, arising from the alternative splicing of three N-terminal exons of the tau gene: exon 2, exon 3, and exon 10. A healthy human brain comprises equal amounts of tau with 3 and 4 repeated microtubule domains. However, some tauopathies show a predominance of isoforms with either 3 or 4 repeated microtubule-binding domains (3R or 4R tauopathies). Other tauopathies, including CTE, are characterized by a roughly equal mix of both 3 and 4 repeated microtubule-binding domains (3R+4R tauopathies)”. In the observations of McKee et al. [15], early-stage CTE (Stage I) is often asymptomatic. Symptoms like headaches, attention deficits, difficulty concentrating, depression, and irritability may occur but are not conclusive indicators. Patients in Stage II CTE may exhibit short-term memory issues, aggressive conduct, explosive reactions, mood fluctuations, and challenges with planning and organization, along with paranoia and suicidal tendencies observed in 27% of cases. As individuals progress to Stages III and IV, cognitive decline and memory loss become apparent, with dementia almost certain by Stage IV. At this stage, distinguishing between CTE dementia and the clinical manifestations associated with AD and FTD may become clinically challenging. McKee and colleagues characterize CTE pathology as “... distinguished by a notable pattern of progressive brain atrophy alongside the accumulation of hyperphosphorylated tau neurofibrillary and glial tangles, dystrophic neurites, 43 kDa TAR DNA-binding protein (TPD-43), neuronal and glial aggregates, microvasculopathy, myelinated axonopathy, neuroinflammation, and white matter degeneration”. Clinical manifestations include behavioral modifications and a gradual, insidious progression of cognitive impairment over decades in some cases. More comprehensive data, including precise timelines of critical events, are necessary to document the incidence and prevalence of PTSD and CTE, as well as genetic risk factors, and the age, circumstances, and nature of past head trauma experienced by CTE sufferers. Among the many challenges linked to TBI, identifying why an acute TBI sometimes leads to CTE remains paramount. Understanding this transition to a chronic state requires pinpointing the steps involved in the progression from an acute to a chronic condition. To tackle the concerning shift from TBI to CTE, Table 1 was compiled to facilitate an analytical comparison of specific trauma-induced disturbances proposed by various researchers as potentially responsible for the TBI to CTE progression. Given that CTE is classified as a tauopathy with a neuropathology defined by “a distinct perivascular accumulation of hyperphosphorylated tau in neurons and astrocytes within cerebral sulci”, it is logical to initially focus on the mechanisms thought to trigger the unexpected emergence of CTE following one or more closed head injuries. Currently, the mechanisms driving the transition from the clinical effects of a single concussion or a series of “mild” TBIs to the severe chronic condition known as CTE are not well understood. Veech et al. [30] suggest that “TBI causes both cerebral blood flow and metabolic impairments, leading to reduced cerebral oxygen uptake, increased lactate production, and depletion of high-energy phosphate reserves in the brain”. Indeed, as reported by T.C. Glenn et al. [31], “the extent of the deficit in cerebral energy metabolism following TBI is the most reliable predictor of outcomes”. Veech et al. [30,31] highlight that cyclosporine A (an early immunosuppressive medication) alleviates TBI’s negative effects in humans and animal models of TBI. They propose that “since cyclosporine has been found to specifically bind to mitochondrial cyclophilin-D and thereby close the mitochondrial permeability transition pore (mPTP), this finding, along with other data regarding MPTP’s role in mitochondrial metabolism, strongly indicates that the opening of this pore initiates the complex series of acute and chronic pathologies associated with TBI”. Moreover, TBI can lead to insulin resistance [30,31], which diminishes the activity of pyruvate dehydrogenase, a crucial step in converting pyruvate into acetyl CoA, necessary for the Krebs cycle’s effective functioning. This cycle generates the reducing power essential for ATP synthesis via the electron transport chain. Consequently, the insulin resistance induced by TBI hampers the Krebs cycle’s efficiency, contributing to central nervous system hypometabolism.

The main clinical and pathological entities are summarized in Table 1 [32].

For the purpose of this review, TBI and concussion contribute both to CTE, and they will not be distinguished as drivers of neurodegeneration and vascular disease.

## 3. Traumatic Brain Injury and Dementia

The question of whether a single moderate-to-severe TBI can trigger late-onset dementia remains debated. As AD accounts for 60–80% of all dementia cases, much of the research on TBI and dementia risk has focused on its relationship with AD. Several studies have found no significant link between TBI and AD development [33,34,35,36,37]. However, other studies suggest that TBI can increase AD risk, with relative risk estimates ranging from twofold to as high as fourteenfold [37]. Two major meta-analyses have examined this association. The first, analyzing 11 case–control studies, and the second, reviewing 15 case–control studies, found that TBI increases AD risk by 58–82%, with males being disproportionately affected [5,38]. Given that women generally have a higher baseline risk for AD [39], the protective effect observed in females following TBI remains an open question requiring further study. Beyond AD, TBI has also been linked to other forms of dementia. A study of World War II Navy and Marine veterans followed 548 individuals with documented TBI and 1228 with non-brain injuries over a 50-year period. The findings revealed that moderate and severe TBIs significantly increased the likelihood of developing both AD and non-AD dementias [40], reinforcing the idea that TBI is a risk factor for multiple types of neurodegeneration rather than AD alone. Similarly, a retrospective cohort study of 164,661 patients aged 55 and older found that moderate-to-severe TBI elevated dementia risk across various subtypes, with a minimum hazard ratio of 1.3 [4].

More targeted investigations have examined the link between TBI and FTD. A retrospective case–control study of 80 FTD patients and 124 controls found that TBI increased the odds of developing FTD by 3.3 times [7]. A separate study of 845 veterans reported an even stronger association, with an odds ratio of 4.4 [41]; however, notably, this study did not find a similar link between TBI and AD. A large-scale study analyzing 147,510 patients from the Taiwanese Longitudinal Health Insurance Database further confirmed these findings, showing that individuals with skull fractures and intracranial injuries had a 4.13 times greater likelihood of developing FTD within four years of TBI [42]. The risk was particularly pronounced in patients under 65, who were at least six times more likely to develop FTD compared to age-matched controls [43].

Together, these studies suggest that while the relationship between TBI and AD remains contested, TBI is increasingly recognized as a risk factor for a broad range of neurodegenerative conditions. Further research is needed to refine our understanding of the mechanisms underlying these associations and to identify potential protective factors that may mitigate the long-term impact of brain injuries.

The likelihood of developing dementia after experiencing a TBI is influenced by a variety of internal and external factors, such as injury severity, patient age at the time of injury, survival duration, genetic predispositions, and cognitive resilience. Research involving World War II Navy and Marine veterans has demonstrated a correlation between TBI severity and dementia risk. Specifically, mTBIs occurring 50 years prior showed no increased risk, whereas moderate TBIs doubled the likelihood of developing AD or other dementias, and severe TBIs quadrupled the risk [40]. Given the extended period between injury and dementia onset in the Plassman study, patient stratification based on age at the time of injury could provide a clearer understanding of risk factors. Supporting this idea, earlier findings indicate that AD risk escalates when the interval between the last TBI and disease onset shortens [43]. A meta-analysis further highlights this trend, revealing that TBIs occurring more than a decade before AD symptoms emerge are associated with a relative risk of 1.63, whereas those occurring within a 10-year window before disease onset carry a significantly higher relative risk of 5.33 [38]. A similar pattern is observed with mTBI, where no increased dementia risk is noted in individuals under 65, but those aged 65 and older face a 20% higher risk [4].

Beyond external risk factors, genetic predispositions also play a crucial role in dementia development following TBI. The presence of the APOE4 gene alone heightens the risk of AD [44], and when combined with TBI, the likelihood of developing the disease surpasses expected levels [45]. However, some studies challenge this association [46], highlighting the need for further investigation into the interplay between TBI and genetic susceptibility.

## 4. Traumatic Brain Injury and Amyloid-Beta Pathology

A shared feature of many neurodegenerative disorders is the abnormal aggregation, misfolding, and accumulation of proteins in the brain. In AD, amyloid-beta (Aβ) plaques and tau tangles are hallmark pathologies [47], while α-synuclein accumulates in PD [48], and TDP-43 is linked to FTD and ALS [49]. Although the role of these proteinopathies in disease initiation remains debated, their presence reflects disrupted cellular processes and brain dysfunction.

TBI, both acute and chronic, frequently features abnormal protein accumulation associated with neurodegenerative diseases. Acute TBI brains exhibit Aβ, tau, and α-synuclein pathologies [50], while chronic cases also show TDP-43 deposition [15,51]. This review focuses on Aβ and tau pathologies after TBI.

Aβ accumulation after TBI has been extensively studied in humans and animal models, highlighting the complex neurodegenerative pathways triggered by injury. The first report of Aβ plaques in severe TBI came from a 1991 postmortem study of 16 cases, which found diffuse Aβ-positive plaques in 38% of injured brains. Interestingly, four out of six plaque-positive cases involved patients over 50, an age group already prone to amyloid deposition [52,53]. A subsequent 1994 study of 152 TBI cases confirmed these findings, showing Aβ deposition in 30% of TBI brains. Notably, individuals aged 51–60 had a 60% incidence of amyloid plaques compared to 20% in those under 50 [54]. These results suggest that aging increases susceptibility to amyloid deposition post-TBI.

The speed of Aβ plaque deposition post-TBI was demonstrated by Ikonomovic et al. (2004) in a study of 18 severe TBI patients [55]. Aβ plaques were detected in cortical tissue within just 2–12 h of injury in 33% of cases, including patients in their 30s. This rapid deposition highlights the accelerated amyloid dynamics triggered by trauma.

Long-term studies have also confirmed Aβ pathology in TBI survivors. Analysis of patients who survived 1–47 years post-TBI revealed Aβ plaques in 30% of cases. Although this matches the prevalence in controls, TBI brains showed higher plaque density and more fibrillar Aβ deposits, indicative of more advanced amyloid pathology [56].

These findings underscore the potential of Aβ as a biomarker for TBI-related neurodegeneration. However, neuropathological studies are limited to static snapshots of brain pathology. Advanced imaging technologies, such as positron emission tomography (PET) with amyloid-specific tracers like Pittsburgh compound B (PiB), offer dynamic insights. For example, PiB imaging in TBI patients showed elevated cortical and striatal Aβ binding within a year of injury compared to controls [55,57]. Future longitudinal imaging studies could clarify whether acute Aβ plaques are cleared, or if TBI accelerates long-term amyloid deposition and neurodegeneration.

Although amyloid plaque formation is observed in only about 30% of severe TBI cases, evidence suggests that intracellular accumulation of amyloid-beta (Aβ) is far more prevalent. A study examining cortical tissue resected from 18 living TBI patients found intracellular Aβ staining in 80% of cases [57], indicating that non-plaque Aβ species are more commonly present post-TBI than extracellular plaques. Similarly, Aβ accumulation in axons is widespread in injured brain samples, regardless of whether plaques are detected [2,58]. Furthermore, prefibrillar Aβ oligomers—high-molecular-weight aggregates—have been identified in the cerebrospinal fluid of some TBI patients within 72 h of injury [59]. Collectively, these findings suggest that while TBI triggers an increase in Aβ peptides in the brain, full aggregation into plaques occurs only in a subset of individuals.

One likely source of this abnormal Aβ accumulation is increased production within damaged axons [3,50]. Amyloid precursor protein (APP) has been found to accumulate along injured axons in regions of diffuse axonal injury in human brains, persisting for up to three years post-injury. APP also co-localizes with β-secretase (BACE1) and the presenilin subunit (PS1) of γ-secretase, the key enzymes responsible for Aβ production [3]. Notably, studies comparing Aβ40 and Aβ42 antibodies indicate that Aβ42 is the dominant species accumulating in injured axonal bulbs [50].

Animal models have consistently replicated these post-TBI amyloid pathologies [3,60,61], providing valuable insights into the mechanisms driving Aβ accumulation. In preclinical studies, elevated levels of BACE1, PS1, and APP—proteins involved in Aβ production—have been observed in the injured cortex following TBI, with expression patterns mirroring the time course of Aβ accumulation [61]. Additionally, experimental models in swine [3,62,63,64] and mice [64] confirm that injured axons serve as focal points of Aβ production after trauma. Importantly, pharmacological inhibition of APP-processing enzymes has been shown to prevent post-TBI Aβ accumulation [61,65], reinforcing the idea that increased Aβ production—rather than reduced clearance—is the primary driver of acute amyloid pathology following TBI.

Despite substantial evidence from human and preclinical studies indicating increased amyloid-beta (Aβ) production after TBI, amyloid plaques are notably absent in the majority (70%) of postmortem TBI brains [55]. This discrepancy suggests that certain factors influence Aβ accumulation and aggregation following TBI (Table 2). 

Although TBI-induced plaques share some characteristics with AD plaques—such as the predominance of Aβ42 [18,55]—they also exhibit key differences (Table 3).

The variability in Aβ plaque deposition following TBI highlights the complex interplay of age, survival time, and genetic predisposition in shaping post-injury amyloid pathology. While TBI clearly promotes Aβ accumulation, its progression to plaque formation appears to be modulated by factors influencing Aβ clearance, particularly neprilysin and APOE4. Understanding these mechanisms could inform targeted therapeutic strategies to mitigate amyloid pathology and long-term neurodegenerative risks in TBI patients.

### 4.1. Tau Pathology in TBI

Tau pathology has been extensively studied in both acute and chronic phases following single and repetitive TBI, whose differential features are summarized in Table 4.

CTE is primarily considered a tauopathy, but co-pathologies are common. A study of 114 neuropathologically confirmed CTE brains found that 52% also exhibited Aβ plaque deposition [76], and additional reports have identified TDP-43 and α-synuclein inclusions in CTE cases [15].

There are both similarities and distinctions between tau pathology in TBI and AD (Table 5).

Developing an animal model that accurately replicates CTE-related tau pathology has been difficult. While acute tau phosphorylation is observed in severe TBI models—similar to TBI-induced Aβ accumulation—chronic tau pathology remains challenging to induce (Table 6).

Despite these efforts, the precise mechanisms driving chronic tau accumulation after repeat mTBI remain unknown. Key unresolved questions include the following:

- Whether a certain injury velocity or location is required to initiate chronic tau aggregation.
- The number of impacts necessary to trigger tau pathology.
- Practical issues such as skin deflection, tissue necrosis, and repeat anesthesia, which complicate model development.

Summarizing, the study of tau pathology after TBI has revealed acute hyperphosphorylation, particularly in axons, but chronic tau accumulation remains less well understood. Evidence from human TBI cases suggests that age, injury severity, and repetitive head impacts all contribute to long-term tau pathology. However, major gaps remain in our understanding of the biochemical and structural properties of post-TBI tau, as well as the exact conditions necessary to induce chronic tau pathology in experimental models. Overcoming these challenges will be critical for developing targeted interventions for TBI-related neurodegeneration.

### 4.2. Polypathology in TBI

While numerous human studies have investigated discrete neurodegenerative pathologies following TBI, there remains a significant gap in the systematic analysis of multiple pathologies within individual cases across large TBI and control populations. Existing research is often limited to single-pathology analyses or descriptive case studies of mixed pathology. A large-scale controlled study examining multiple neurodegenerative markers in postmortem TBI brains could provide critical insights into factors influencing diverse neuropathological phenotypes after TBI and help explain the polypathology observed in both acute and chronic TBI cases [15] (Table 7).

A comprehensive approach would provide insights into how these variables interact to shape the long-term neuropathological landscape of TBI survivors.

A key question in TBI research is whether the presence of neurodegenerative disease pathology in a subset of post-TBI patients contributes to increased dementia risk. Does the accumulation of pathological proteins drive further neurodegeneration or accelerate brain aging? Evidence suggests that neuritic Aβ plaques in CTE brains are associated with advanced disease staging and dementia [70]. This raises the possibility that Aβ deposition may act as a catalyst for disease progression following repetitive mTBI.

Beyond pathology itself, several factors may modulate dementia risk in TBI survivors [18,51,54,56]:

- Age at the time of injury: Younger individuals may have greater resilience, while older individuals may be more vulnerable to long-term neurodegeneration.
- Duration of survival post-injury: Longer survival times allow for the development and progression of neuropathology.
- Repeated head trauma or increased injury severity: More frequent or severe injuries may increase the likelihood of developing neurodegenerative pathology.
- APOE4 genotype: The presence of the APOE4 allele is a known risk factor for Alzheimer’s disease and has been linked to worse outcomes following TBI.

To fully understand the relationship between TBI, neuropathology, and dementia risk, it is essential to establish large, well-controlled cohorts of TBI brains. These studies should aim to dissect the interplay between injury characteristics, genetic predisposition, and neuropathological burden in determining long-term cognitive outcomes. By addressing these knowledge gaps, researchers can refine strategies for early intervention, risk stratification, and therapeutic development for TBI-related neurodegeneration.

## 5. Traumatic Brain Injury and Cerebral Amyloid Angiopathy

CAA is a cerebrovascular condition marked by the accumulation of beta-amyloid (Aβ) in the walls of cerebral arteries, arterioles, and capillaries [64,83]. This pathology is prevalent in older adults and is detected in up to 98% of AD cases [84]. Aging and genetic predispositions, particularly the presence of the APOE ε4 allele, significantly influence CAA development [83,85,86]. The condition predominantly affects leptomeningeal and superficial intracortical vessels, with a higher occurrence in the posterior cerebral cortex, particularly the parietal and occipital lobes, compared to the frontal lobes [80,81].

Aβ deposition progressively weakens vessel walls, increasing their susceptibility to rupture and leading to intracerebral hemorrhage (ICH), making CAA a primary cause of ICH in the elderly [84,87,88,89]. Beyond hemorrhagic events, CAA is linked to cognitive decline and dementia, likely due to a combination of hemorrhagic, ischemic, and synaptic damage mechanisms [75,90].

Emerging research suggests a connection between TBI, repetitive head impacts (RHIs), and neurodegenerative diseases [4,14,17,91]. Contact sports such as American football, boxing, and ice hockey are associated with an increased risk of CTE. Notably, even subconcussive impacts—head injuries that do not produce overt symptoms—can result in neuronal damage [15,92]. The duration of contact-sport participation correlates with the severity of tau pathology and the pathological stage of CTE [15,17,93,94]. In American football, head impacts frequently involve the anterior skull, with biomechanical studies indicating that the greatest strain occurs in the frontal convexities and deep sulci [70,95,96]. These regions align with the earliest and most severe tau pathology in CTE and suggest that RHI may contribute to a frontal distribution of CAA and other related pathologies [15,17].

While studies on single TBI have not established a significant link to CAA [97,98], the relationship between RHI and small-vessel disease remains poorly understood. Notably, amateur football players exhibit blood–brain barrier (BBB) dysfunction, as demonstrated by dynamic contrast-enhanced MRI following a single season of play compared to non-contact-sport athletes [99]. This finding raises the possibility that RHI predisposes individuals to small-vessel vascular abnormalities, including CAA.

An individual study addressed this issue, starting from the hypothesis that subjects with CTE or a history of RHI would exhibit an altered distribution, increased prevalence, and greater severity of CAA [100]. In order to confirm this hypothesis, the authors analyzed 807 autopsies from three different studies: (1) the Understanding Neurological Injury and Traumatic Encephalopathy (UNITE) group (357 subjects with a history of exposure to contact sports such as football, ice hockey, boxing, soccer, rugby, and martial arts at either the professional or amateur level) [27]; (2) the Boston University’s Alzheimer’s Disease Center (BUADC) (241 subjects) [101]; and (3) the Framingham Heart Study (FHS) (209 subjects). In this study, CAA was evaluated across four cortical regions: the dorsolateral frontal lobe (Brodmann areas 6 and 8), superior temporal lobe (BA 20, 21, and 22), inferior parietal lobe (BA 39 and 40), and calcarine cortices (BA 17 and 18). The assessment and scoring of CAA were conducted following the methodology outlined by Vonsattel et al. on a semi-quantitative scale from 0 to 3, based on the extent of Aβ deposition within blood vessels using Aβ immunohistochemistry [102]. Leptomeningeal and intracortical vessels were scored separately, and a global CAA severity score was established according to NIA-AA guidelines [103]. The findings revealed that the prevalence of CAA was highest in the AD (95.7%), and CTE and AD groups (81.8%) compared to the no-CTE/no-AD (54.3%) and CTE (28.7%) groups. A hierarchical cluster analysis of the pathological data (presence and grading of CAA) indicated that cluster 2 exhibited significantly more severe disease in the leptomeninges across all regions compared to the cortex (** *p* < 0.001). The proportion of individuals with CTE was significantly greater in cluster 2 compared to clusters 1 and 4 (*p* < 0.001), while clusters 1 and 4 had a notably higher percentage of individuals with AD than cluster 2 (*p* < 0.001). Regarding the distribution of CAA, participants with AD showed a significantly higher frequency of CAA in the parietal lobe in both the cortex and leptomeninges compared to the frontal lobe. In contrast, participants with CTE exhibited increased CAA frequency in the leptomeninges of both the frontal and parietal lobes compared to the cortex (* *p* < 0.05, *** *p* < 0.001, χ^2^ tests, b). Participants with AD also had elevated CAA severity in the parietal lobe (including both cortex and leptomeninges) compared to the frontal lobe compartments. Conversely, those with CTE showed increased CAA severity in the leptomeninges of the frontal lobe compared to the frontal or parietal cortices. CAA severity was significantly higher in the frontal lobe of participants with CTE compared to AD in the leptomeninges (*p* < 0.001) and trended toward increased CAA in the frontal cortex (*p* = 0.052). Additionally, CAA severity was significantly elevated in the leptomeninges of participants with CTE compared to those with AD in both the frontal and parietal lobes (*p*’s = 0.002). For parts b-d, * *p* < 0.05, ** *p* < 0.01, and *** *p* < 0.001. CAA was linked to alterations in Aβ, tau pathology in the sulci, and synaptic density. Aβ1-40; Aβ1-42; tau pathology (AT8 density); and PSD-95, a marker of synaptic density, were quantified via immunoassay in the dorsolateral frontal cortex, revealing that Aβ1-40 was significantly increased in those with both CTE and CAA compared to those with CTE alone. Aβ1-42 levels were significantly higher in individuals with both CTE and CAA, as well as in those without CTE or AD. Tau pathology, assessed through AT8 density, was significantly elevated within the sulcal depths in participants with both CTE and CAA compared to those with CTE only. CAA was associated with a notable decrease in age-adjusted means of PSD-95 density in participants without CTE or AD, as well as in those with CTE. According to this analysis, the CAA severity score showed a grade 3 in a significantly higher proportion of AD (22.7%), and CTE and AD group (22.2%). In addition, the CTE and AD subgroup had a more severe pathological degree of CAA (stage IV 59.1% vs. 23.1% in CTE). Both AD and CTE and AD subgroups had LBD in the neocortex significantly more frequently than the other ones (14.3% and 13.6%, respectively).

In AD, comparisons of CAA between leptomeningeal and intracortical vessels showed no differences in frequency; however, CAA severity was found to be lower in the leptomeninges compared to the cortex. In contrast, in CTE, CAA frequency was significantly higher in both the frontal (*p* < 0.001) and parietal (*p* = 0.015) leptomeninges. Additionally, CAA severity was elevated in the frontal leptomeninges compared to both the frontal and parietal cortices (*p* = 0.010). While there were no significant differences in CAA severity between the superior temporal and calcarine cortices in CTE, cases of AD demonstrated greater CAA severity in the superior temporal gyrus compared to the calcarine gyrus. Notably, similar regional distribution patterns were observed when participants were classified based on their exposure to contact sports rather than pathology. Individuals with a history of contact sports exhibited CAA frequency and severity patterns resembling those of the CTE group, while those without such exposure showed patterns akin to the AD group. Direct comparisons between AD and CTE emphasized these distribution differences. The difference in CAA severity between the frontal and parietal lobes (Δfrontal–parietal) was significantly greater in CTE than in AD within the leptomeninges (*p* < 0.001) and trended toward significance in intracortical vessels (*p* = 0.052). Similarly, the difference in CAA severity between leptomeningeal and intracortical vessels indicated significantly increased leptomeningeal CAA in CTE compared to AD in both the frontal and parietal lobes (*p* = 0.002). In regard to the relationships between neuropathological diagnoses, repetitive head injury (RHI) from contact sports, and CAA, a distinct group with leptomeningeal-dominant CAA was identified, primarily composed of individuals with CTE and a history of contact-sport participation. Further pathological analysis revealed that CTE cases exhibited greater CAA involvement in leptomeningeal vessels than in intracortical vessels, with a preference for the frontal lobe. In contrast, AD cases had more CAA in the parietal lobe than the frontal lobe across both leptomeningeal and intracortical vessels. Ordinal logistic regression analysis showed that while contact-sport history was not significantly associated with overall CAA presence, it was linked to more severe CAA in the frontal leptomeninges, even after adjusting for age, APOE ε4, and AD. Additionally, CAA presence correlated with increased neurodegenerative proteins, reduced PSD-95 levels, and dementia. While contact-sport participation did not appear to increase CAA risk, it influenced its severity and distribution when present. Postmortem analyses of individuals with a single TBI revealed BBB disruption and extravasated serum proteins in brain tissue years after injury [91,104], findings also noted in a CTE case [105]. Other studies suggest an increased Aβ burden in individuals with prior TBI [106]. Animal models have demonstrated TBI-related meningeal cerebrovascular damage, including intravascular leakage into CSF [107,108,109] and significant BBB disruption following repetitive concussive and blast exposures [104,110]. However, a pooled autopsy study found no association between TBI (with or without loss of consciousness) and overall CAA presence, though it did not assess CAA distribution or severity [97].

CAA distribution has been widely studied in aging populations, primarily in relation to cortical-region involvement [86,87]. In AD, CAA is most prevalent in the parietal and occipital lobes [64,86,87,111,112], often favoring leptomeningeal over intracortical vessels [4,6]. Our findings confirm that AD cases had greater CAA severity in the parietal lobe than the frontal lobe. In contrast, CAA in CTE was more severe in the frontal lobes and was predominantly leptomeningeal, independent of age at death. The susceptibility of leptomeningeal vessels to RHI-related strain may stem from their tethering to the pia mater and positioning within sulci [70]. Cortical capillary involvement represents a distinct CAA subtype associated with APOE ε4 [113,114], while dysphoric CAA has been linked to tau pathology [7]. Though leptomeningeal CAA predominated in RHI and CTE cases, without prominent dysphoric angiopathy, further research is needed to assess capillary CAA and its association with RHI and CTE.

One functional consequence of CAA may be the loss of vessel elasticity, impairing perivascular clearance of solutes from brain parenchyma into CSF [109,115,116]. As a result, CAA may contribute to Aβ and tau protein accumulation in the brain. In CTE cases, CAA was associated with significantly elevated Aβ1-40 and Aβ1-42 levels, sulcal tau pathology, and reduced PSD-95 expression (a synaptic density marker). While CAA-related vessel dysfunction may drive tau accumulation, tau pathology itself could also contribute to vessel damage and subsequent CAA development [116]. Future studies should further explore the temporal and spatial relationship between CAA and tau accumulation in CTE.

## 6. Pathophysiological Issues

The primary pathology of TBI is driven by mechanical forces that result in immediate cellular damage, including cell lysis; axonal disconnection; and the release of intracellular ions, neurotransmitters, and proteins. Severe and penetrating brain injuries lead to significant neurovascular dysfunction, disrupting the BBB and allowing the influx of peripheral proteins and immune cells into the brain parenchyma. This disruption is particularly critical in severe TBI cases. In contrast, the effects of repetitive rmTBIs are subtler but still manifest as axonal and vascular pathologies.

Following the primary injury, secondary-injury processes develop over a longer timescale, including oxidative stress, excitotoxicity, activation of cell death pathways, and neuroinflammation. In an aging brain, these secondary injury processes become exacerbated, and recovery mechanisms such as synaptogenesis and angiogenesis are compromised. The implications are significant, as these secondary injury effects may create essential mechanistic connections between TBI and AD, especially in older individuals. Genetic and epigenetic factors can further modify these processes, influencing the risk of developing AD later in life.

### 6.1. Diffuse Axonal Injury

Diffuse axonal injury (DAI) is a significant characteristic associated with TBI. The mechanical forces involved disrupt the intra-axonal cytoskeletal structure and enhance axolemmal permeability, resulting in a pathological influx of Ca2+ ions. This triggers the activation of caspases, calpain-mediated spectrin proteolysis, and mitochondrial damage [58]. Histologically, axonal swellings, which are a hallmark of DAI, manifest as axonal bulbs (retraction balls) that accumulate cellular organelles, vesicles, and proteins. Among the proteins typically involved in fast axonal transport, APP accumulates within hours after TBI in these axonal swellings, co-localizing with Aβ [117]. Consequently, axonal APP immunoreactivity is considered a reliable marker of DAI [86,118] and appears prior to the emergence of silver-staining positivity, which signifies neuronal or axonal degeneration [119]. Additionally, axonal APP immunoreactive axonopathy has been noted in long-term survivors of blast TBI, characterized by spheroids and varicosities with a perivascular distribution in cortical white matter [118]. The interaction between axonal injury and APP/Aβ is likely intensified with aging [2]. The relationship between axonal accumulation of APP/Aβ and parenchymal Aβ plaque deposition following TBI remains unclear. APP-immunoreactive deposits are commonly found in areas of axonal injury [55], and swollen axons that show APP immunoreactivity are associated with Aβ plaques, particularly during the acute phase after TBI [55]. This indicates that injured axons may play a role in contributing to the Aβ found in plaques. However, a study involving individuals with remote brain injuries noted an absence of widespread plaque pathology despite the accumulation of Aβ in axons, leading researchers to suggest that plaques induced by TBI might regress over time [2]. Notably, APP/Aβ deposition is more frequently observed in older individuals who experience a TBI, often resulting from falls, which are the leading cause of head injuries in the elderly [120]. Consequently, advanced age may exacerbate AD pathological processes following TBI.

Recent advancements in imaging techniques have greatly improved the ability to detect and monitor axonal injury and Aβ deposition in living patients with TBI. For example, MRI methods such as diffusion tensor imaging (DTI) and high-definition fiber tractography [121] have been utilized to evaluate axonal integrity. Additionally, amyloid PET imaging has provided significant insights into Aβ accumulation [122]. These clinical analyses often corroborate findings from histopathological studies. For instance, Kawai et al. [123] reported a 27% retention of [C-11]PiB in imaged patients, reflecting the proportion of severe TBI cases that show histological evidence of Aβ plaques. Similarly, another study indicated that mild cognitive impairment (MCI) subjects undergoing [C-11]PiB PET imaging at the Mayo Clinic Study of Aging exhibited greater PiB-PET retention when they self-reported a history of TBI [105]. Furthermore, in a study of long-term survivors of moderate-to-severe TBI, increased PiB-PET retention was found to correlate with DTI evidence of altered connectivity in the posterior cingulate cortex [124], a region particularly susceptible to Aβ accumulation in Alzheimer’s disease. It is essential to utilize these imaging modalities alongside assessments of vascular dysfunction to evaluate the relationship between amyloid lesions and cardiovascular disease following TBI, especially in aging brains, as has been investigated in non-TBI subjects.

### 6.2. Synapse Loss

Synapse loss represents a critical outcome of TBI. DAI disrupts axonal transport, resulting in disconnection and deafferentation [125]. The progression of this phenomenon differs between experimental models and humans and is linked to synaptic alterations [126]. Despite the extensive axonal damage that can occur post-TBI, it has been highlighted that “the number of degenerating nerve terminals significantly surpasses the number of identified damaged fibers” [125], underscoring the importance of synapse degeneration in the context of TBI. The identification of synaptic degeneration or loss following TBI can be complicated by ongoing restorative mechanisms, such as synapse regeneration, which may represent a compensatory response or an inappropriate reaction to synaptic loss. Experimental research has indicated that this regenerative process is active in the hippocampus after brain injury [127] and can be influenced by age. For example, in a model of entorhinal cortex lesions, older rats exhibited a greater reduction in synapse density in the outer molecular layer of the dentate gyrus compared to younger rats [128]. Synapse loss acts as a structural correlate of cognitive decline in AD and may heighten the risk of developing dementia after TBI, especially when combined with aging. In clinical populations without cognitive impairments, as well as in those with MCI and mild AD, synapse loss occurs progressively in specific neocortical regions, such as the inferior temporal gyrus and posterior cingulate cortex [129]. Conversely, in certain areas like the precuneus, synapse loss is only evident in mild AD [129]. Chronic synapse loss following experimental TBI may reflect the regional vulnerability patterns observed in AD. For instance, cortical impact injuries can lead to significant synapse loss in the hippocampus, a region particularly susceptible to synaptic degeneration in AD [129]. The involvement of Aβ in synapse loss after experimental TBI is yet to be completely understood. Controlled cortical impact (CCI) injury resulted in a more pronounced loss of pre- and post-synaptic markers in human Aβ (hAβ) knock-in mice compared to wild-type mice exhibiting endogenous (mouse) Aβ [130]. This finding suggests that human Aβ oligomers may contribute to synaptic degeneration following TBI, akin to mechanisms seen in AD [130]. Advanced age, particularly in individuals over 65, is associated with synapse loss, potentially contributing to the late-onset dementia often observed in older TBI survivors, as significant synaptic loss may lower the threshold for the clinical manifestation of AD.

### 6.3. Oxidative Stress

Oxidative stress is a significant factor that emerges in the aftermath of TBI. Reactive oxygen species (ROS), which are generated from mitochondrial leakage or the accumulation of Aβ, are believed to contribute to oxidative damage and cellular dysfunction, particularly in aging and AD [131]. Research conducted by Scheff et al. highlights a connection between oxidative stress and the loss of synaptic proteins in relation to Aβ pathology in both preclinical and prodromal stages of AD [132]. Although the causal relationship between ROS and synaptic dysfunction in neurodegenerative conditions remains a topic of debate, it is likely that both factors influence outcomes, including the risk of developing AD after TBI [133]. Following TBI, ROS production often increases, possibly leading to DNA damage, protein and lipid peroxidation, mitochondrial dysfunction, and ultimately cell death [134]. At the neurovascular-unit level, excessive ROS can induce cell death and compromise the integrity of the BBB. Aging is associated with mitochondrial dysfunction and diminished levels of antioxidant enzymes, which can elevate oxidative stress within the neurovascular unit, partly through the formation of peroxynitrite from the interaction of nitric oxide derived from endothelial cells and superoxide. The presence of Aβ may exacerbate these mechanisms or, conversely, promote the accumulation of Aβ in the brain. The relationship between Aβ and oxidative stress following TBI was explored in a study involving nine-month-old Tg2576 mice (prior to plaque deposition) and their wild-type counterparts subjected to repetitive mild TBI (rmTBI). This research revealed increased concentrations and deposition of Aβ, heightened levels of lipid peroxidation, and cognitive deficits in the transgenic mice, while these effects were not observed in the wild-type group [135]. These findings suggest that the accumulation of Aβ in the brain and oxidative stress may work together to enhance the risk of AD following TBI [133,135]. Overall, ROS appear to play an active role in the pathology of TBI, aging, and AD, indicating that targeting oxidative stress could be a promising therapeutic strategy to mitigate the connection between brain injury and the development of AD [132,136,137].

### 6.4. Apoptosis

Apoptosis plays a crucial role as a mechanism of cell death in the context of TBI. Pro-apoptotic factors, such as cytochrome c and apoptosis-inducing factor, are released from damaged mitochondria, activating caspases in injured axonal segments and contributing to cytoskeletal damage. The increased production of Aβ following TBI may further promote apoptosis through caspase activation, which enhances the amyloidogenic processing of APP. This heightened caspase cleavage of APP after TBI or during aging can lead to an overproduction of Aβ, potentially establishing a link between TBI and the pathogenesis of AD [138]. Interestingly, Koliatsos et al. found that individuals with no cognitive impairment (NCI) exhibited minimal-to-no evidence of apoptosis in the absence of Aβ pathology. However, apoptosis was present in NCI cases with Aβ plaques—often an early indicator of preclinical AD—and was significantly elevated in brains affected by AD. Although this area warrants further investigation, these findings suggest that TBI patients who develop Aβ plaques may face increased neuronal loss, and with advancing age, this could further decrease the threshold for the clinical onset of AD. In our studies, we indirectly assessed the impact of caspase-cleaved APP and human Aβ on outcomes following TBI [139]. We observed increased levels of APP, caspase-cleaved APP, and Aβ monomers/oligomers following severe CCI injury in human Aβ (hAβ) knock-in mice. Notably, the immediate inhibition of pan-caspases after CCI injury led to reduced levels of both caspase-cleaved APP and Aβ, resulting in improved histological outcomes [139]. This indicates that targeting caspases in the aftermath of TBI may help alleviate injury-induced AD-like pathology. Furthermore, aging may exacerbate the apoptotic response to brain injury, negatively affecting neurological recovery.

### 6.5. Neuroinflammation

Neuroinflammation is a significant factor in the aftermath of TBI. Aging is linked to a shift toward a pro-inflammatory state, which is characterized by increased microglial reactivity and microglial senescence [140]. The presence of microgliosis and reactive astrocytosis coincides with amyloid lesions in AD and may contribute to Aβ-related neurodegeneration following TBI. After TBI, astrocytic signaling pathways become activated, resulting in various morphological changes that depend on the severity of the injury [141]. These observations suggest a connection between injury-induced changes in Aβ and the activation of astrocytes following TBI, potentially aiding in the clearance of cellular debris and facilitating neurovascular remodeling [142]. This process involves various receptors, including toll-like receptors (TLRs) and the receptor for advanced glycation end-products (RAGEs). Astrocytes are capable of internalizing and metabolizing Aβ, possibly through interactions with low-density lipoprotein receptor-related protein 1. In the aftermath of TBI, astrocytes also release inflammatory mediators such as cyclooxygenase-2 and metalloproteinases, which are implicated in AD and may play a role in sequestering toxic Aβ. Microglia can exert both protective and harmful effects following TBI, depending on their polarization into M1 (pro-inflammatory) or M2 (immunosuppressive) phenotypes [139]. The different activation states of microglia and their associated neurorepair versus neurotoxic processes warrant further investigation, particularly concerning changes in Aβ following TBI. In adult human Aβ (hAβ) knock-in mice subjected to CCI injury, we observed a rapid (within hours) and sustained (over several weeks) increase in microglia immunoreactive for ionized calcium-binding adaptor molecule 1. The progression of this reactive response aligned with increased accumulation of human Aβ and a loss of synaptophysin immunoreactivity in both the cortex and hippocampus [140]. Recent findings suggest that TBI and aging may jointly influence microglial function. Aging appears to modify microglial activity post-TBI, potentially leading to morphological changes that could impact recovery at various stages. In aging brains, microglial profiles shift to a primed inflammatory state characterized by an exaggerated cytokine response (such as IL-1β) and increased expression of inflammatory (IL-1β and TNF-α) and immune (MHC II) markers, akin to conditions seen after TBI and in AD [141]. Moreover, aging impairs the microglial capacity to internalize Aβ, even though they maintain the ability to adhere to Aβ fibrils in situ (in plaques) and in vitro [140]. This age-related decline in Aβ clearance may help clarify the heightened vulnerability to neurodegenerative processes following injury in older individuals. Animal studies have also shown that peripheral monocyte infiltration and activation are significantly increased in aged mice after TBI [139]. Consequently, the inflammatory processes triggered post-TBI may be intensified by age-related pathophysiology, ultimately raising the risk of developing Alzheimer’s disease later in life [141,142].

### 6.6. Loss of Pyramidal Cells

Finally, both blunt head injury in humans and AD share the loss of pyramidal cells in specific subfields of the hippocampus, and this could be a further issue triggering neurodegeneration after TBI [143]. In fact, this study investigates the differential loss of neurons in various hippocampal sub-fields following severe blunt head injury in humans. Using stereological methods, the researchers measured neuron counts in the CA1, CA2, CA3, and CA4 sub-fields of the hippocampus across three patient groups: early survivors (1 week or less), late survivors (6 months or more), and controls (age-matched individuals with no head injury). The results showed the following: (1) significant loss of neurons in CA1, CA3, and CA4 within 1 week of injury; (2) no neuron loss in CA2 across all groups; and (3) continued neuron loss in CA1 and CA4 between 1 week and 6 months after injury, but no loss in CA2 or CA3. Then, the study concluded that severe head injury leads to an initial acute loss of neurons in all hippocampal sub-fields except CA2, with ongoing neuron loss in CA1 and CA4 in longer-surviving patients. CA2 remains unaffected throughout [143].

## 7. Structural Neuroimaging Markers

MRI atrophy patterns have become instrumental in diagnosing and monitoring neurodegenerative diseases such as AD and FTD [142,144]. The rates of atrophy are utilized as endpoints in large-scale multi-center clinical trials assessing disease-modifying therapies [141] and may also offer similar potential in trials focused on post-traumatic neurodegeneration [142]. Recently, in vivo MRI investigations have begun to uncover structural signatures in individuals at risk for CTE, employing automated techniques and hybrid imaging analysis to derive quantitative volumetric indices. In a study conducted at UCSF, voxel-wise analyses indicated that participants with TES exhibited reduced volumes in the lateral and medial frontal cortices, insula, and anterior temporal lobes when compared to age- and education-matched amyloid-negative individuals without a history of RHI [145]. Some TES participants showed signs of cortical thinning and subcortical gray matter volume loss, with the most pronounced decrease occurring in the hippocampus. Another study focused on frontotemporal regions, comparing 19 former Canadian Football League players (mean age = 50; age range = 30–74 years) to age- and education-matched healthy males without a concussion history, revealing diminished thickness in the left anterior temporal lobe, but not in the orbitofrontal cortex [146]. A separate investigation analyzed MRI-derived cortical thickness in 11 former NCAA Division I American football players (mean age = 27; age range = 24–32; scanned an average of four years post-football participation) versus 10 demographically similar track-and-field athletes. The football players demonstrated reduced cortical thickness in various regions, including the left frontal pole and bilateral temporal gyri [145]. Although the hippocampus was not examined, no significant differences were found in the parahippocampal gyrus or other frontal and temporal lobe regions. One volumetric MRI study assessed limbic system structures in 86 symptomatic former NFL players (mean age = 54.86) compared to 22 asymptomatic same-age men without a history of RHI or TBI [147]. The results indicated smaller bilateral volumes of the amygdala, hippocampus, and cingulate gyrus in former NFL players compared to their asymptomatic peers. In another investigation, volume loss was analyzed across 14 regions of interest in nine former NFL players (all over 55 years) and nine age-matched cognitively normal controls [148]. The former players exhibited greater volume loss in the right hippocampus after adjusting for multiple comparisons, while no differences were noted in other regions. Decreased hippocampal volume has also been reported in other small cohorts of aging symptomatic former NFL players [149]. Notably, Misquitta et al. [150] found that interactions between age and exposure led to a more pronounced effect of age on hippocampal volumes in 53 former Canadian Football League players (mean age = 55.6) compared to 25 age- and education-matched males without a concussion history, as well as 321 age-matched males from the Cambridge Centre for Aging and Neuroscience. A sample of 34 retired fighters from the Professional Fighters Brain Health Study (PFBHS) (mean age = 47) showed reduced volumes in the hippocampus, amygdala, and thalamus compared to 62 age-matched individuals without a TBI history [151]. However, no differences were observed in the caudate, putamen, or total brain volume. Longitudinal MRI studies involving individuals exposed to RHI are still limited. Bernick et al. examined changes in brain volume over time among 173 active and retired professional fighters from the PFBHS in comparison to individuals without RHI exposure [152]. This sample included 23 retired boxers, 50 active boxers, 100 active mixed martial artists, and 31 cognitively normal individuals without RHI history. Participants underwent annual assessments, with all having at least two MRIs over a mean follow-up period of approximately 2-3 years. Retired boxers displayed an accelerated decline in the left and right amygdala and right hippocampus. These findings should be considered alongside existing null studies. Coughlin et al. presented additional data comparing 14 NFL players (4 active and 10 retired, ages 24–39) with 16 age-, sex-, education-, and BMI-matched controls across various brain regions, including the hippocampus and amygdala [153]. No significant group differences were reported. The goals of this manuscript did not include these comparisons, and the relatively young age of the sample may account for these null volumetric effects. Zivadinov et al. [154] compared 21 former NFL and NHL players (mean age = 56) with 21 age-matched non-contact-sport athletes, finding no significant differences in global or regional brain volumes. However, their regional analyses were limited, raising concerns about the potential suppression of effects by the former NHL players. Another study found minimal evidence of chronic brain injury in a sample of 45 former NFL players, with atrophy observed in only 2 participants [155]. This research lacked a comparison group and was primarily descriptive. Bang et al. reported no structural MRI differences between five retired professional boxers (mean age = 46.8) and four age-matched controls [156]. An additional study compared volumetric regions between 20 former high school football players with two or more mild TBIs (ages 40–65) and 20 matched former players without a lifetime history of TBI [157]. No group effects were identified. The implications of these null findings remain uncertain, particularly given that all participants played American football. Other research has similarly explored neuroimaging indices among former university-level contact-sport athletes, yielding varied effects and interpretations regarding concussion versus RHI exposure [158,159].

Evidence concerning the corpus callosum (CC) in aging individuals at high risk for CTE is limited. A longitudinal study by Bernick et al. within the Professional Fighters Brain Health Study (PFBHS) found that retired boxers exhibited accelerated shrinkage of the CC compared to their control group [160]. Additionally, a sample of 34 retired fighters showed lower CC volumes when compared to 62 age-matched individuals without a history of TBI [161]. A DTI study analyzing CC integrity in 34 former NFL players (ages 41–79, both symptomatic and asymptomatic) compared to 85 matched asymptomatic individuals revealed that symptomatic former NFL players (n = 14) had reduced fractional anisotropy (FA) in the CC compared to their matched controls (n = 14) [162]. No significant effects were observed in the asymptomatic former NFL players. The presence of a cavum septum pellucidum (CSP) is not exclusive to CTE, as it is a common finding in the general adult population [163]. Autopsy-confirmed cases of CTE often reveal abnormalities in the septum pellucidum [155,164], and in vivo MRI studies support these findings [15,165]. In a UCSF study involving 11 participants with traumatic encephalopathy syndrome (TES), 8 were found to have a CSP, with widths ranging from 3 to 11 mm and lengths from 1.5 to 4.5 mm [166]. Among 39 retired fighters from the PFBHS and 63 age-matched individuals without TBI history, 77% of the retired fighters had a CSP, and 51% had a cavum vergae, compared to 17% and 0% in the comparison group, respectively (*p* < 0.01) [27]. The CSP and cavum vergae were also found to be longer in retired fighters. Similar results emerged from a study involving 72 symptomatic former NFL players (mean age = 54.53) and 14 asymptomatic men of the same age without a history of RHI or TBI, where 92% of former players and 57% of their counterparts had a CSP (*p* < 0.01), with CSP length being greater in the former NFL players. Another investigation involving 17 symptomatic former professional football players (mean age = 54.6) reported higher rates of CSP compared to 17 age- and sex-matched individuals without a history of TBI (94% vs. 18%) [27]. The CSP was found to be of a higher grade and longer in length among former professional football players. FLAIR MRI is frequently used to assess the presence and severity of white-matter hyperintensities (WMHs), which are believed to have a vascular origin and are associated with aging and vascular risk factors such as hypertension and diabetes [91,148,167,168]. In dementia evaluations, FLAIR WMHs are often utilized to assess contributions from cerebral small vessel disease to cognitive or neuropsychiatric impairments, aiding in the diagnosis of vascular cognitive disorders (e.g., vascular dementia) [155]. Former elite contact-sport athletes may be at increased risk for FLAIR WMHs due to a higher prevalence of vascular risk factors in this demographic [169,170,171,172,173,174,175]. These WMHs are non-specific and may also indicate various white matter neuropathologies related to RHI exposure, including white-matter rarefaction, neuroinflammation, axonal loss, demyelination, and gliosis. Hart et al. [163] found greater total (mean difference = 5.75) and deep (mean difference = 0.79) FLAIR WMHs in 10 cognitively impaired former NFL players (mean age = 66.6) compared to 20 age-, estimated-IQ-, and education-matched asymptomatic participants without a concussion history or involvement in college or professional football. However, no statistically significant group effect was found for periventricular WMHs. A study involving 86 symptomatic former NFL players (mean age = 54.86) compared to 23 asymptomatic same-age men without RHI or TBI history showed that former players had increased volumes of T1-weighted WM hypointensities after controlling for age and total brain volume [176]. Both groups exhibited similar vascular risk scores, based on a modified Framingham Stroke Risk Profile, but a limitation of this study was the reliance on T1-weighted scans for estimating WM lesion volume rather than FLAIR. Zivadinov et al. [155] reported no differences in the total number or volume of WM signal abnormalities between 21 former NFL and NHL players (mean age = 56) and 21 age-matched non-contact-sport athletes. The evaluation of white-matter lesions utilized semi-automated methods applied to T2/PD/FLAIR images. SWI/T2-weighted GRE sequences are routinely used to detect cerebral microbleeds, which may indicate underlying cerebrovascular disease associated with cognitive and neuropsychiatric decline, and can aid in diagnosing CAA [177]. Microbleeds have been identified as neurological sequelae of TBI and active RHI [174,178,179], potentially implicating them in CTE, especially given the associations between RHI exposure and microvascular pathology [93,180,181]. In neuroimaging studies on high-risk participants for CTE, cerebral microbleeds have not been a primary focus, and when reported, they have been infrequently observed [153,161,182,183], consistent with minimal occurrences in neuropathological examinations of deceased American football players [93].

Advanced neuroimaging techniques have significantly enhanced our understanding of individuals at high risk for CTE. Studies utilizing translocator protein 18 kDa (TSPO) PET imaging have revealed increased inflammation in this population [149,154]. Additionally, DTI has shown greater white-matter injury, particularly within frontotemporal tracts [91,160,163]. Functional MRI studies have identified deficits in brain activation and connectivity [99,184,185], while neurochemical alterations have been observed through magnetic resonance spectroscopy. Furthermore, reductions in global and regional cerebral blood flow have been assessed using arterial spin labeling MRI [163,186]. Dynamic contrast-enhanced (DCE) MRI has indicated disruptions to the blood–brain barrier [187], and electrophysiological changes have been captured by electroencephalography (EEG). DTI, in particular, has been extensively studied in the context of RHI since diffuse axonal injury is a central pathology associated with TBI [188]. Ex vivo DTI analyses of CTE tissue have demonstrated a correlation between fractional anisotropy and axonal disruption in white-matter regions that co-localize with phosphorylated tau (p-tau) pathology associated with CTE [189]. Despite the valuable insights provided by DTI, it requires prolonged scan acquisition times and complex post-processing. The clinical relevance of DTI-derived indices, such as fractional anisotropy, remains uncertain, especially in relation to neurodegenerative diseases. Consequently, DTI and similar advanced imaging sequences are not routinely used in dementia evaluations due to the lack of FDA approval for indications in AD and other age-related dementias (ADRDs). Nonetheless, ongoing research utilizing these neuroimaging techniques will continue to shed light on disease pathogenesis, underlying mechanisms, and associated neuropathologies.

## 8. Discussion

CTE has currently been well described as a tauopathy, supported by both postmortem histopathological findings and in vivo imaging. Its association with the deposition of multiple pathological proteins, including beta-amyloid, has been demonstrated. However, the aspect that has been most frequently explored is the parenchymal neurodegenerative side, rather than the vascular side. Specifically, even when focusing on post-traumatic amyloid-related pathology, TBI has been conceived and proposed as a driver of neurodegeneration (AD side) rather than vascular disease (CAA side). In reality, although not frequently investigated, CAA has been documented in association with CTE, as described in previous sections. This suggests that it is still largely an unexplored context, but one that exists, and the main challenges lie in formulating and testing a diagnostic hypothesis in vivo. Indeed, the diagnostic criteria for CAA require that micro- and macrohemorrhagic findings have no explanation other than CAA^11^. TBI is a sufficient cause for superficial cortical siderosis and microbleeds. The use of cerebrospinal fluid biomarkers and PET imaging with amyloid tracers has not been standardized in this field, and their diagnostic impact is currently difficult to assess.

**Figure 1:**
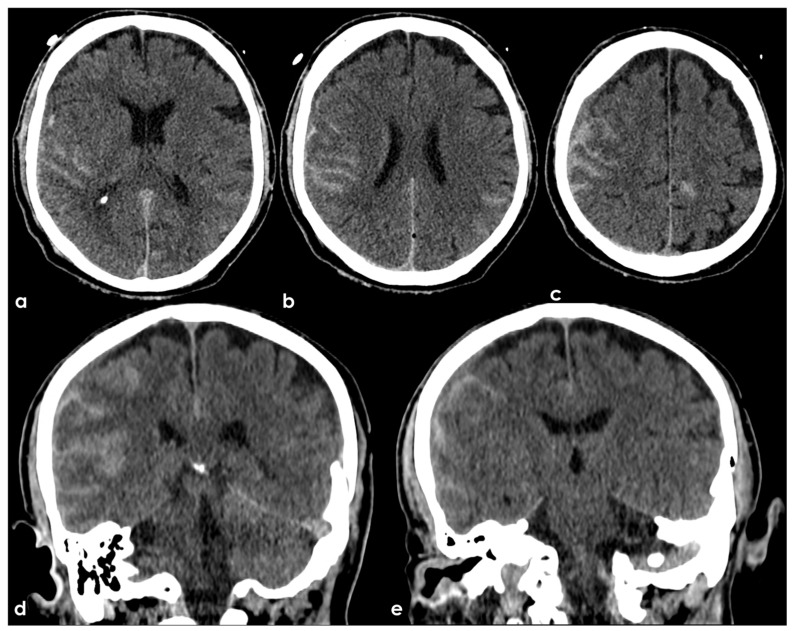
NCCT three hours after an accidental fall from a staircase. Panels (**a**–**c**) show the axial slices and panels (**d**,**e**) the coronal slices with bilateral hyperdense filling of several subarachnoid sulci as for bleeding and slightly hypodense contusion and edema of the adjacent brain parenchyma.

In this regard, we believe that an example from clinical practice can help define the largely unexplored area in which we find ourselves. Figure 1 shows the brain non-contrast computed tomography (NCCT) of a 68 years-old man after an accidental fall from a staircase. His past medical history was uneventful, and the brain did not show any significant abnormality apart from the post-traumatic subarachnoid bleeding and the concussion.

**Figure 2:**
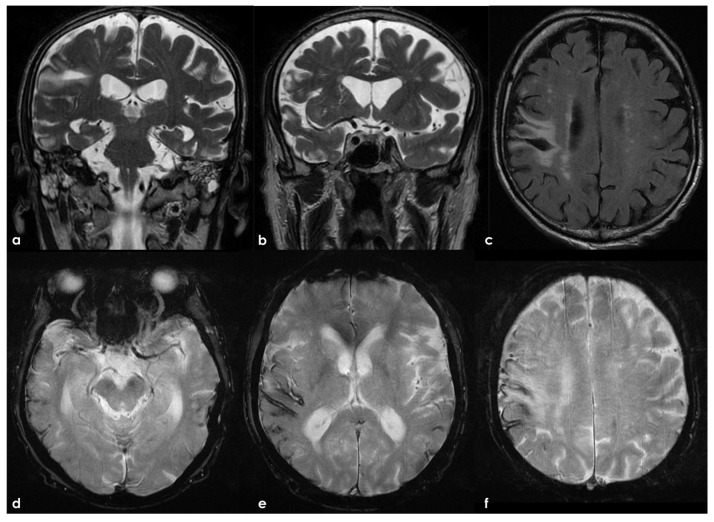
Brain MRI 6 months after the TBI, showing in T2W coronal images (panels **a**,**b**) and in FLAIR axial images (panel **c**) the scar of the previous contusion and a visually significant brain atrophy. Conversely, in GRE images (panels **d**–**f**), a multifocal cortical superficial siderosis emerges, mainly in the right hemisphere.

The patient underwent a brain MRI 8 months after the TBI (Figure 2), showing atrophy, the evolution of post-traumatic parenchymal damage, and a diffuse cortical superficial siderosis; when in the acute phase, subarachnoid bleeding was documented.

**Figure 3:**
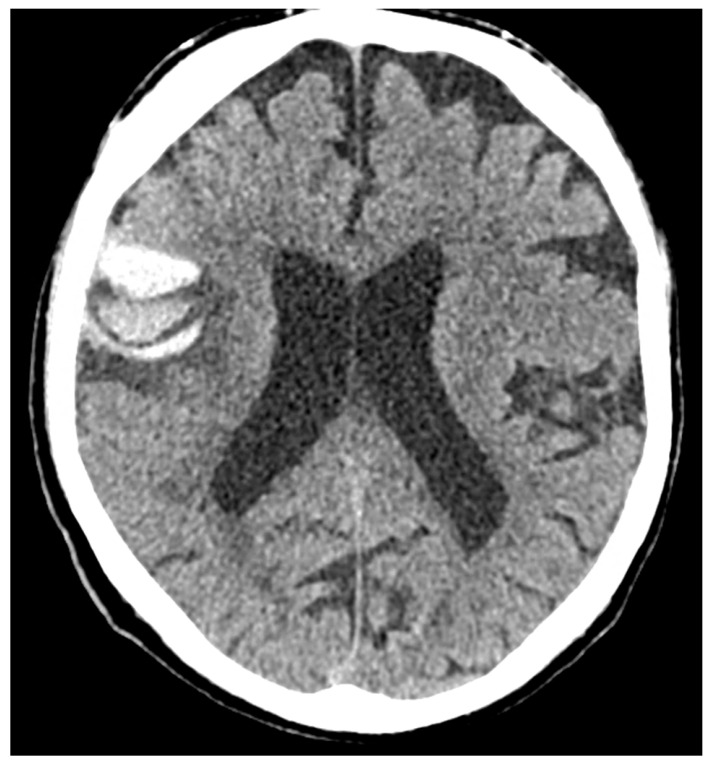
Brain lobar hemorrhage with different densities (blend sign) in the right frontal lobe (NCCT, axial slices).

The same patient went to the hospital because of the sudden onset of a seizure, performing an NCCT with a spontaneous lobar hemorrhage in the right frontal operculum, near the previous contusion (Figure 3). CT angiography and catheter angiography were performed during hospital stay, with unremarkable findings.

**Figure 4:**
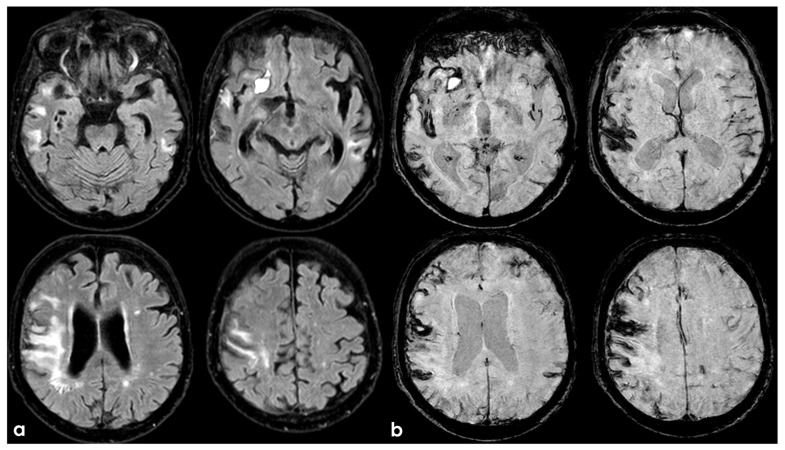
Brain MRI showing axial FLAIR (panel (**a**)) and axial Susceptibility Weighted Imaging (SWI) minimum intensity projection (panel (**b**)) slices. The new hemorrhage was hyperintense on both sequences, and the assed cortical superficial siderosis was well identifiable in comparison with previous MRI.

The brain MRI performed after 30 days showed a new hemorrhage in the right frontal basal region, with satellite cortical superficial siderosis, and with new siderosis in the right frontal lobe in the left hemisphere (Figure 4).

**Figure 5:**
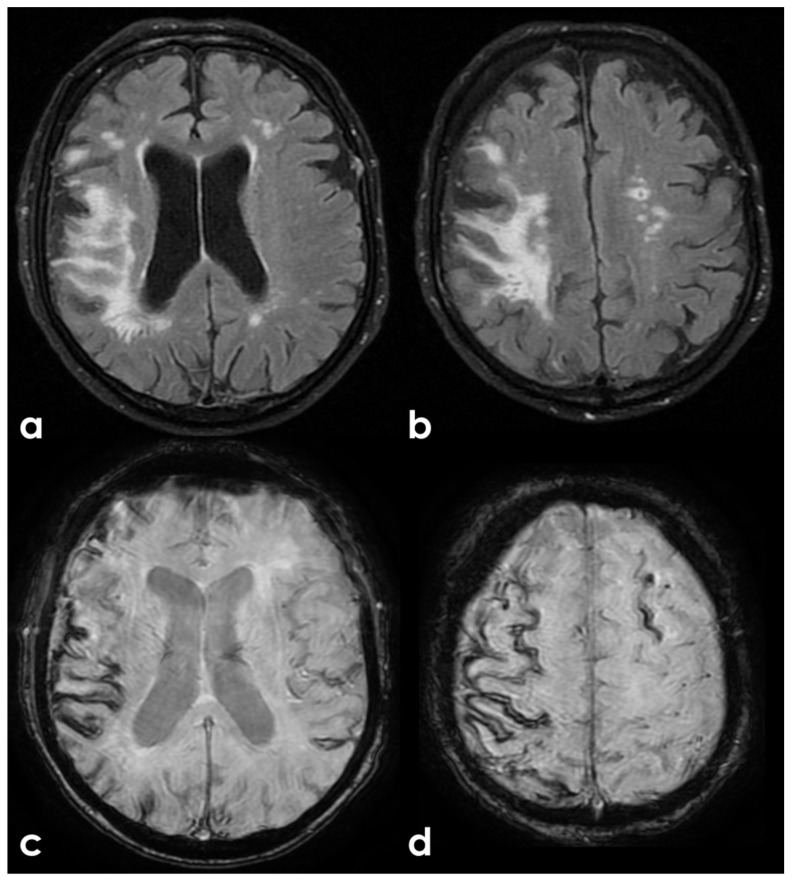
Control MRI, showing an increasing in white matter hyperintensity ion the right hemisphere (axial FLAIR, panels (**a**,**b**)) and an increased burden of cortical superficial siderosis (SWI in panels (**c**,**d**)) in the left superior frontal sulcus.

A follow-up MRI after 12 months showed a further increase in cortical superficial siderosis in the left hemisphere (Figure 5).

**Figure 6:**
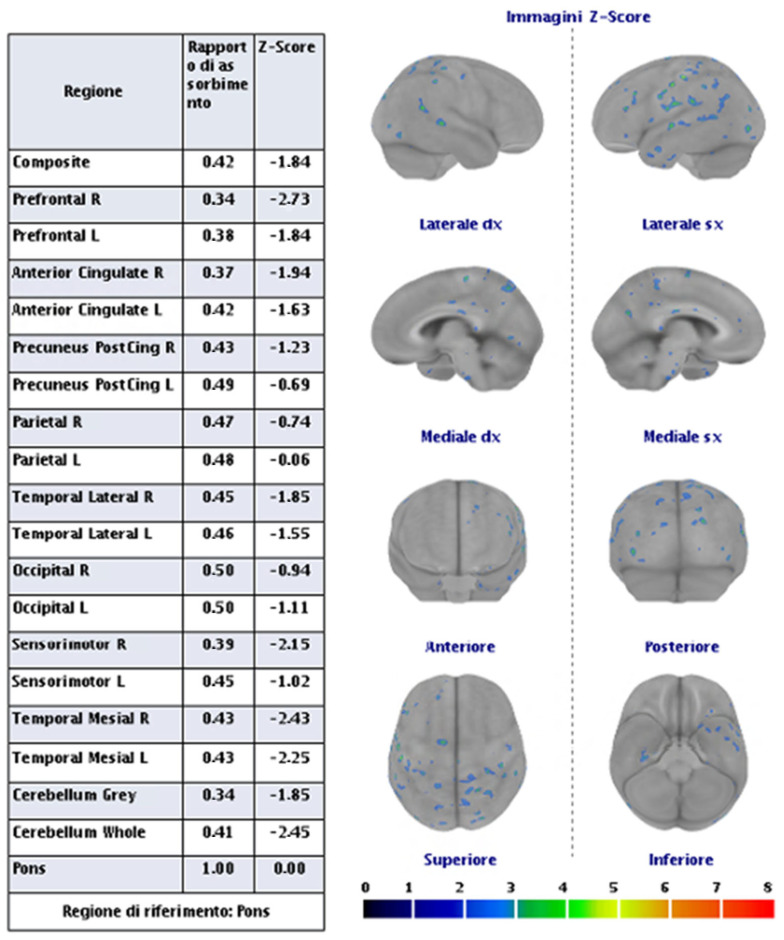
PET with amyloid tracer shows the absence of significant cortical capitation of the tracer.

The patient underwent ApoE genotype testing (E37E3), lumbar puncture for neurodegeneration markers dosage, and PET with Flumetamol (Figure 6). The CSF biomarkers were as follows: Aß42, 397 pg/mL (725–1777); Aß40, 4374 pg/mL (7755–16,715); Aß42/40, 0.090 pg/mL (0.068–0.115); Tau, 132 pg/mL (146–410); and pTau181, 17.8 pg/mL (21.5–59.0).

Ultimately, the presented case has characteristics that, in the absence of the previous traumatic history, would have been diagnostic for probable CAA. However, the prior traumatic event prevents the formulation of this diagnosis without histopathological evidence meeting the currently validated criteria. Furthermore, in the present case, no suggestive elements for an AD pattern were identified.

## 9. Conclusions

In conclusion, the relationship between TBI and CAA remains complex and underexplored, highlighting the need for further research to clarify their associations. While TBI is known to contribute to neurodegenerative processes, distinguishing between TBI-related pathologies and those characteristic of CAA poses significant diagnostic challenges. The interplay between vascular changes and neurodegeneration emphasizes the necessity for comprehensive diagnostic criteria and advanced imaging techniques. Ultimately, a better understanding of these interactions could lead to improved clinical outcomes and targeted therapeutic strategies for individuals affected by TBI and related neurodegenerative conditions.

## Figures and Tables

**Table 1:** Main clinical and pathological entities in cerebral traumatic injury.

Definitions	Features
Traumatic brain injury (TBI)	Both loss of consciousness (LOC) and post-traumatic amnesia (PTA) should be present in symptomatic cases. The duration of LOC or PTA, and the Glasgow Coma Scale score are used to grade the severity (“mild”, “moderate”, or “severe”, depending on. This framework is more frequently applied in studies involving civilian populations in emergency department settings or among military personnel and veterans, rather than in cases of sports-related head trauma.
Concussion	It is considered synonymous with mild TBI. However, in sports contexts, concussion diagnoses are frequently established on the presence of head trauma and headaches, dizziness, impaired balance, nausea, or abnormalities in eye movement, but not LOC or PTA.
Subconcussive trauma	It is a common occurrence in collision sports (e.g., American football) as asymptomatic head impacts Similarly, in military environments, service members might face subconcussive exposure due to repeated blast incidents or training exercises (such as breacher or combat training).
Traumatic encephalopathy syndrome (TES)	Research criteria have been proposed for classifying cognitive and neuro-behavioral symptoms thought to be linked to repetitive head trauma, with symptoms often emerging years after the last exposure to head trauma. They have high sensitivity but low specificity regarding the underlying neuropathology of chronic traumatic encephalopathy (CTE).
Chronic traumatic encephalopathy (CTE)	The consensus diagnostic criteria about neuropathological findings include phosphorylated tau protein aggregates located in neurons around blood vessels at the depths of cortical sulci. The diagnosis of CTE is established independently of the patient’s symptoms during their lifetime.

**Table 2:** Factors affecting the Aβ accumulation and aggregation following TBI.

Age and survival time	Two key factors affecting Aβ deposition post-TBI are the age at the time of injury and the length of survival. The incidence of Aβ plaques is higher in older patients after acute severe TBI [52,54], and longer survival appears to correlate with more extensive and mature Aβ pathology [4]. This suggests that, given sufficient time, Aβ deposition may progress from diffuse accumulations to more structured plaques.
Genetic variations in Aβ clearance	Neprilysin: - It degrades Aβ in the brain, and polymorphisms in the gene can influence Aβ plaque deposition after TBI [66]. - Specific neprilysin alleles, such as those with extended GT repeats, are associated with an increased risk of Aβ plaques, while other polymorphisms are linked to a decreased risk [66]. - In long-term TBI survivors, neprilysin accumulates within axonal bulbs, co-localizing with APP and Aβ, suggesting a role in local Aβ clearance [66]. - Neprilysin levels are reduced in patients with CTE compared to non-demented controls [67], further implicating impaired clearance in Aβ accumulation.
	Apolipoprotein E (APOE): - It is a key protein in Aβ clearance. - The gene has three common alleles: APOE2, APOE3, and APOE4. - The APOE4 allele is associated with impaired Aβ clearance from the brain [68]. Retrospective analysis of cases from Roberts et al. [69] found that Aβ plaque deposition after TBI occurs more frequently in APOE4 carriers. This effect appears to be dose-dependent: Only 10% of individuals without APOE4 (5 of 50 cases) developed plaques. 35% of heterozygous APOE4 carriers (12 of 34 cases) developed plaques. 100% of homozygous APOE4 carriers (6 of 6 cases) developed plaques. - In preclinical studies, mice expressing human APOE4 show greater post-TBI amyloid deposition, including fibrillar Aβ deposits [54] and increased intracellular Aβ accumulation from 1 week to 12 weeks post-injury compared to APOE3-expressing mice [70]. - Genetic variations affecting Aβ clearance may predispose certain individuals to greater Aβ accumulation and aggregation after TBI.

**Table 3:** Differences between TBI-induced plaques and AD plaques.

Plaque morphology	Aβ deposits in TBI brains are more diffuse than the dense-core neuritic plaques typically seen in advanced AD [52,54,55]. This suggests that post-TBI plaques form rapidly and recently, rather than undergoing the prolonged maturation process seen in AD.
Neuritic plaques	While neuritic plaques have been identified in some TBI brains, they are primarily found in older individuals [52,54]. Thioflavin-S staining—used to detect β-sheet structures in mature amyloid plaques—was positive in only 1 of 18 acute TBI cases [55]. In long-term TBI survivors, a higher prevalence of fibrillar Thioflavin-S-positive plaques has been reported [4], suggesting that plaque maturation may require extended post-injury survival.

**Table 4:** Summary of the main differences of tau pathology in different stages of TBI.

TBI Stages	Features
Acute tau pathology after single TBI	Within 24 h of injury, phosphorylation of tau at the Ser396/Ser404 epitope is observed in axons and white matter of excised TBI brain tissue [71]. However, somatodendritic tau accumulation is rare, suggesting that while hyperphosphorylation occurs acutely, neurofibrillary tangle formation does not. Only 11% of acute postmortem TBI brains show p-tau immunoreactivity, and tau-positive glial cells are present in up to 20% of severe TBI cases.
Chronic tau pathology in long-term TBI survivors	A study of 39 severe TBI cases with survival times ranging from 1 to 47 years compared to 47 control brains found tau pathology in 34% of TBI brains aged under 60, compared to only 9% of controls [72]. Additionally, the distribution of tau pathology in TBI brains differed from controls, with abnormal tau staining appearing in sulcal depths and superficial cortical layers, rather than being restricted to the entorhinal cortex and hippocampus, as seen in control brains. Widespread tau pathology was detected in the cingulate gyrus, superior frontal gyrus, and insular cortex.
Repetitive mTBI and CTE	Most reports of tau pathology post-TBI come from cases of repetitive mTBI associated with CTE [73]. Initially, CTE was primarily documented in boxers. However, in recent years, multiple cases have been identified in athletes from various contact sports, as well as military personnel with a history of blast and military-related concussions [74,75].

**Table 5:** Comparison of TBI-associated tau pathology and AD.

Issues	Features
Morphology	Tau-positive somatodendritic inclusions in CTE resemble those in AD, but CTE features a significantly greater degree of astrocytic tau deposition.
Neocortical distribution	In long-term single-TBI survivors and CTE cases, tau preferentially accumulates in layers II and III of the cortex [4], whereas in AD, tau deposition is typically concentrated in layers V and VI [77].
Biochemical characterization	Limited studies have examined the biochemical profiles of tau post-TBI. The inclusion type (paired helical filaments, straight, or ribbon filaments), primary phosphorylation sites, and roles of different tau isoforms remain unclear. Only two studies have assessed the ratio of 4-repeat (4R) to 3-repeat (3R) tau in CTE. One study found that both isoforms were hyperphosphorylated in brain extracts from two boxers [78]. Another reported both 4R and 3R tau immunostaining in a human CTE case, with 4R tau predominantly found in astrocytic tau inclusions [15]. However, no biochemical analyses have been conducted on the chronic tau pathology observed in long-term survivors of a single TBI.

**Table 6:** Challenges in modeling tau pathology after TBI.

Issues	Features
Acute tau phosphorylation	In 3xTg-AD mice (harboring a human P301L tau mutation), cortical impact injury led to punctate, primarily axonal, p-tau accumulation across multiple brain regions, as well as increased somatodendritic p-tau in contralateral CA1 neurons. Endogenous mouse tau phosphorylation at multiple sites has been observed following blast injury [78,79] and closed-head injury.
Chronic tau accumulation	Inducing sustained tau pathology in animal models has been difficult, even with tau transgenic mice. In one study using T44 mice (overexpressing the shortest tau isoform), animals received four mTBIs per day, once a week for four weeks (16 impacts total), followed by a 9-month recovery period. Only one mouse exhibited accelerated tau deposition [80]. Another study using aged (18-month-old) human tau (hTau) mice subjected to five mTBIs over nine days found increased tau pathology three weeks post-injury compared to sham or single-mTBI mice [81].

**Table 7:** Main issue on polypathology of TBI.

Issues	Features
Incidence and overlooked pathologies	Current studies that focus on individual pathological markers report that amyloid-β (Aβ) and tau pathology are present in approximately 30% of TBI cases. However, the true incidence of neurodegenerative pathology post-TBI could be considerably higher if multiple disease-related proteins—such as TDP-43, α-synuclein, and phosphorylated tau—were examined systematically within the same cohorts [9,70]. A broader investigation would provide a more accurate understanding of the pathological burden following TBI.
Animal models and the importance of multi-pathology analysis	Experimental models of TBI could also benefit from simultaneous investigation of multiple neurodegenerative markers within individual animals. This would help determine whether different pathological processes follow distinct temporal progressions after injury and whether certain pathologies are more strongly associated with specific types of injury. Studies by Tran et al. examined both Aβ and tau pathology following cortical impact injury in 3xTg-AD mice, which carry mutations linked to AD [82]. Future studies should incorporate additional factors that influence neurodegenerative pathology phenotypes, including the following ones.
Individual issues	Injury severity; Age at the time of injury; Duration of survival post-injury.
Genetic background	APOE genotype

## Data Availability

No new data were created or analyzed in this study.
